# Impact, feasibility, and acceptability of CREATORS: An arts-based pilot intervention to reduce mental-health-related stigma among youth in Hyderabad, India

**DOI:** 10.1016/j.ssmmh.2024.100339

**Published:** 2024-08-08

**Authors:** Shivani Mathur Gaiha, Antonio Gasparrini, Mirja Koschorke, Usha Raman, Mark Petticrew, Tatiana Taylor Salisbury

**Affiliations:** a Indian Institute of Public Health-Hyderabad, India; b Department of Public Health, Environments and Society, Faculty of Public Health and Policy, London School of Hygiene and Tropical Medicine, UK; c Division of Adolescent/Young Adult Medicine, Boston Children’s Hospital, Boston, MA, USA; d Faculty of Pediatrics, Harvard Medical School, Boston, MA, USA; e Centre for Global Mental Health, Institute of Psychiatry, Psychology & Neuroscience, King’s College London, UK; f Sarojini Naidu School of Arts & Communication, University of Hyderabad, India

## Abstract

**Background:**

Mental-health-related stigma prevents youth from seeking help for mental health problems. Limited studies in low- and middle-income countries assess the effect of arts-based education in reducing such stigma among youth, thereby leaving a gap in evidence-based, age- and culturally-appropriate interventions.

**Objective:**

To evaluate the impact, feasibility, and acceptability of CREATORS, an arts-based educational program on reducing mental-health-related stigma among youth in India.

**Methods:**

We conducted a mixed-methods, pre-post control group study among college-going adolescents in Hyderabad, India. At baseline and post-intervention (after six weeks), we examined differences in intended behavior towards people with mental health problems.

**Results:**

Our study involved 432 participants across three study groups: participants creating art on the theme of mental-health-related stigma over six weeks (n = 123), a student audience viewing 2-h arts show by participants (n = 244), and a control group (n = 65). Between baseline and post-test, participants creating art showed significantly lower stigma towards people with mental health problems compared to members of the control group (coefficient = 1.55, 95%CI 0.06–3.04, p = 0.041). Participants found the intervention useful and enjoyable (>95%; n = 773 across six weeks). Participants identified that collaborative creation of art made the subject of mental health interesting and relatable.

**Conclusions:**

Participating in an arts-based educational program was associated with significantly lower mental-health-related stigma among youth compared to a control group in the short term. High acceptability of the program demonstrates the utility of arts-based education to address mental-health-related stigma. With community partners and artists as facilitators, our program may support mental health specialists in mental health promotion.

## 1 Introduction

Globally, mental health problems are experienced by 10–20% of all children and adolescents (Kieling et al., 2011). Public stigma associated with mental health problems negatively affects help-seeking (Yap et al., 2013). Public stigma comprises problems of knowledge, negative attitudes and discriminatory actual or intended behavior directed towards people with mental health problems (Thornicroft et al., 2007), and is different from internalized or self-stigma experienced by those suffering from mental health problems. Adolescents and young adults are potentially more vulnerable to public stigma given that 75% of all mental disorders are first discovered among people when they are of this age (Kessler et al., 2007) and since evidence suggests that adolescents hold negative views and display and isolating behaviors towards people with mental health problems (Gaiha et al., 2020; Koike et al., 2017; Walker et al., 2008). Reducing mental-health-related public stigma among adolescents and young adults may improve attitudes and behaviors, and, in turn, aid in timely detection and continued social support for individuals experiencing mental health problems and/or diagnosed conditions.

Education and social contact (interactions with people living with mental health problems) are effective strategies to reduce mental-health-related stigma (Corrigan et al., 2012; Thornicroft et al., 2016). Several stigma-reduction interventions focus on mental health problems generally and encourage student-led projects and creative activities (Pinfold et al., 2003; Pittman et al., 2010; Rahman et al., 1998; Rickwood et al., 2004). Entertainment-education or arts-based education for health promotion, using theater, dance, painting, and other art forms, aims to achieve greater interactivity, model empathic behaviors, and appeal to emotions and/or aspirations of youth with the ultimate aim of changing health-related behaviors (Gaiha and Salisbury, 2022). Research indicates that interventions using multiple art forms improve behavior towards people with mental health problems (Gaiha et al., 2021). However, there is a dearth of evidence from youth-focused arts-based interventions to reduce stigma in low- and middle-income countries, like India, where the magnitude of mental-health-related stigma may be comparable to developed countries (Gaiha et al., 2020).

Although most studies about youth in India characterize public stigma (S. M. Gaiha et al., 2020), a community-based anti-stigma campaign used theater to challenge prejudice and social exclusion related to common mental disorders (Maulik et al., 2017). Among college youth in India, a single-group study found that using dance-drama, a PowerPoint presentation, and social contact increased positive attitudes (Ahuja et al., 2017). Given that dance, storytelling, and theater are a firm part of India’s heritage and modern culture (Chakravorty and Gupta, 2012; Junik-Łuniewska, 2019; Karnad, 1989), arts-based educational interventions are potentially well-suited to the context to reduce mental-health-related stigma among adolescent and young adult populations. Further, a chronic shortage of mental health professionals in India begs for community partnerships to expand mental health promotion (Devassy et al., 2022; Shidhaye et al., 2017).

Thus, while the need to reduce stigma among youth in India is well-documented, creative, and educational interventions that transform perceptions and behavior beyond sharing information are lacking. To address this gap, an arts-based educational program to reduce stigma was developed and piloted in India. The aim of this study was to evaluate whether the program reduced stigma among participants relative to a control group, and assess feasibility, and acceptability of the program.

## 2 Methods

### 2.1 Study design and setting

A mixed methods, quasi-experimental pre-test and post-test control group study was used to assess feasibility, acceptability, and impact of a pilot program in Hyderabad, India. This study received ethics committee approval from the London School of Hygiene and Tropical Medicine, UK and the Indian Institute of Public Health-Hyderabad.

### 2.2 Participants and recruitment

Colleges were enrolled through one-on-one meetings with the college principals or management. Participants were recruited through a week-long recruitment drive where the first author and student volunteers displayed the program poster, conducted information sessions and classroom announcements, and distributed consent forms. Participation in the intervention was open to any student who committed to completing all sessions at the start. A subset of participants was invited to focus groups to represent sex and varying roles in the arts-based intervention (e.gs. actor, scriptwriter, creator of visual art, stage setup, etc.). A student audience was recruited by program participants to attend an arts show and members of the control group were recruited by the first author from another college (>20 km away). No exclusion criteria were applied. No participants were required to disclose their mental health status (past or present experience of problems or diagnoses) or caregiver status at any time. All participants provided informed consent prior to joining the study.

### 2.3 Intervention design

CREATORS is an acronym for College cReative Expression using Art to Transform Opinions Regarding mental-health-related Stigma. The CREATORS program was developed based on a pilot awareness intervention (), evidence from anti-stigma-education (Gaiha et al., 2021; Gaiha and Salisbury, 2022), and interviews and an intervention development workshop with 77 individuals (college management and faculty, people living with mental health problems, caregivers, mental health specialists, youth counselors, artists, researchers, and college youth).

CREATORS is a series of six, 2-h sessions held weekly with college youth on the theme of mental health. The program is based on theoretical frameworks related to public stigma (Goffman, 1968; Link and Phelan, 2001; Yang et al., 2007), social learning and health promotion (Ajzen, 1991; Bandura and Walters, 1977), and art/expression promoting learning and social justice (Fisher, 1984; Goldblatt, 2006; Krathwohl, 2002) (see Supplemental Figure 1). The content focuses on breaking myths and stereotypes about mental health problems to enable empathy towards people with mental health problems (see Supplemental Figure 2). Art is used as an educational tool to promote reflection on common manifestations of mental health-related stigma. Activity-based sessions engage participants in three phases of learning: 1) Observe, in which participants observe/react to verbal and visual prompts and social contact; 2) Create, in which participants develop artwork on the theme of mental health problems and related stigma; and 3) Show, in which participants showcase their artwork to an invited audience of students (Supplemental Table 1). Program facilitators included mental health counsellors, artists, and facilitators with a youth-focused community-based organization.

### 2.4 Intervention groups

#### 2.4.1 Participants

Participants include all students who completed sessions as part of the CREATORS program and were involved in developing or exhibiting the arts show. This is our primary group among whom we will examine the impact, acceptability, and feasibility of CREATORS.

#### 2.4.2 Student audience

Members of this group include all students who attended an arts show as part of CREATORS. The student audience was included to expand reach of program messages to a wider group and to enable participants to encounter opinions that challenge their learning about mental-health-related stigma during the program.

#### 2.4.3 Control group

No intervention was delivered to the control group.

#### 2.4.4 Variables and data collection procedures

Pen-and-paper, pre- (baseline) and post-test surveys were completed by participants before the start of session one and within one week after the program was completed (at six weeks), by the student audience at the start of the arts show and six weeks later, and by the control group at recruitment and six weeks later. At baseline, socio-demographic information, participation in extra-curricular activities (yes/no), and whether participants knew someone with a mental health problem (yes/no) were collected.

#### 2.4.5 Impact

The Reported and Intended Behavior Scale (RIBS) is the primary outcome of interest, which measures intended behavior towards people with mental health problems as representative of the extent of public stigma (Evans-Lacko et al., 2011). The scale includes four items on willingness to live with, work with, live nearby, and continue a relationship with people living with mental health problems. Responses are scored from 4 to 20, where a higher score indicates less stigma. As problems of knowledge, attitude and behavior collectively contribute to stigma, participants were asked individual items corresponding to these domains (Evans-Lacko et al., 2010; Griffiths et al., 2008; Taylor and Dear, 1981). Impact data were collected from participants, student audience and control group members at baseline and six-week follow-up.

#### 2.4.6 Feasibility

Attendance registers assessed recruitment and retention at each session. Focus groups with participants and interviews with facilitators were conducted during the week after the program by the first author and a field research assistant; questions included perceptions about collective co-creation of art and barriers and facilitators of participating. At the end of each session, facilitators completed a checklist to identify completed activities, assess time available for discussion, and rate participant engagement.

#### 2.4.7 Acceptability

Participant acceptability was assessed through feedback forms at the end of each session, including items related to: 1) stigma-related content (thought-provoking, ease of understanding, usefulness), 2) using an arts-based format (attention and involvement, entertaining or interesting, ability to share perceptions, feeling an emotional connection with the issue) and 3) experience (energy/atmosphere, discussion, and management of timings and venue). Responses to these items were on a Likert scale from 1 to 4, where 1 indicates strongly disagree and 4 indicates strongly agree. Focus groups assessed participant acceptability through questions about program benefits, harms, strengths, and weaknesses.

#### 2.4.8 Data analyses

In this mixed-methods study, data were triangulated by comparing quantitative and qualitative findings, seeking complementarity, consistency, and explanations on feasibility, acceptability, and impact of the program (Cresswell and Plano Clark, 2011). Qualitative data were analyzed using inductive coding of themes and patterns guided by grounded theory (Braun and Clarke, 2006; Thomas, 2006) till thematic saturation was reached (Saunders et al., 2018), using NVivo 10 Software (QSR International, 2014). Multi-level mixed effects linear regression analysis determined whether there was a significant difference in mean scores on intended behavior (primary outcome) between groups (participant, student audience, and control group) as measured between pre- and post-assessment (time). Covariates included gender, type of previous school, cost of transportation, prior participation in extra-curricular activities, and knowing someone who lives with a mental health problem. Statistical significance was set at p < 0.05. Quantitative analyses were performed using Stata version 14. We provide adapted COREQ (Tong et al., 2007) and STROBE (von Elm et al., 2008) quality checklists corresponding to qualitative and quantitative research in this mixed-methods study (see supplemental material).

## 3 Results

Four-hundred and thirty-two students from five colleges were recruited into the study (see Table 1), including 123 participants attending four or more sessions and creating art (participants). The CREATORS arts-based program engaged participants to create 150+ pieces of art and sculptures, eight theatrical plays, and three choreographed dances. Most participants were female (57.3%), studied Science (56.5%), had not previously participated in extra-curricular activities like theater or art (62.9%), and did not know someone with mental health problem (62.1%). Other students participated as an audience (n = 244) or the control group (n = 65). Across groups, fewer participants knew someone with a mental health problem (34.7%) compared to audience (40.1%) and controls (50.0%).

### 3.1 Impact

#### 3.1.1 Behavior (primary outcome)

Participants’ mean scores on the Reported and Intended Behavior scale improved among participants creating art (2.36 mean score difference) and the student audience (0.91 mean score difference) between pre- and post-test (see Table 2). In the adjusted regression analysis, we observed a significant reduction in stigma measured by improved mean RIBS scores of intended behavior towards people living with mental health problems after 6 weeks (coefficient 1.55, 95% CI = 0.05, −3.04, p = 0.041) among participants creating art compared to the control group (see Table 3). Knowing someone with a mental health problem at baseline made it less likely for an individual’s stigma to reduce at post-test (coefficient 0.85, 95%CI 0.36, 1.33). The Intraclass Correlation Coefficient (ICC) across colleges was 0.023 (standard error 0.02, 95%CI 0.004–0.104). Between pre-test and post-test there was a significant increase in participants creating art who initiated a conversation about mental health (21.0% vs. 30.6%).

#### 3.1.2 Knowledge

Participants showed favorable and significant improvements in knowledge between pre-test and post-test (see Table 2), including that people with mental health problems are less dangerous than most people think (67.7% vs. 70.9%), medication can be an effective treatment for people with mental health problems (67.7% vs. 72.6%), and in the ability to identify the specific location of mental health services (32.3% vs. 61.3%). Two knowledge-related questions showed a decline between pre- and post-test, including that virtually anyone can have poor mental health (81.4% vs. 79.8%) and recognition of specific symptoms of depression based on a scenario/vignette (87.9% vs. 81.4%). There were significant improvements in the student audience’s’ knowledge, except that fewer audience members recognized specific symptoms of depression based on a scenario/vignette (69.7% vs. 68.2) and believed in the efficacy of medication (72.3% vs. 70.1%) at post-test. The control group’s knowledge declined between pre- and post-test (all items).

#### 3.1.3 Attitude

Between pre-test and post-test, participants creating art showed an improvement only on 3 of 5 attitude-related questions, perceiving that people with mental health problems are not a burden on society (76.6% vs 78.2%), that mental health problems are real medical illnesses (30.6% vs. 36.3%), and not a sign of weakness (20.2% vs. 24.2%) (see Table 2). The student audience showed an improvement on attitude-related questions, including that people with mental health problems are not a burden on society (72.7% vs. 80.3%), that it is best not to avoid people with a mental health problem (58.7% vs. 63.2%), and that people can overcome mental health problems (77.6% vs. 82.6%). The control group improved in two attitude-related questions, i.e., that mental health problems are real medical illnesses (22.1% vs. 26.7%) and are not a sign of weakness (9.3% vs. 11.6%).

### 3.1.4 Feasibility

The CREATORS program was feasible to implement since participants were able to create art on the theme of mental-health-related stigma and nearly all activities went as planned. Some feasibility issues were experienced. Specifically, in all colleges, college management asserted that the program remain open to walk-ins from participants (regardless of session) and so 61% more participants were recruited than 24–36 planned per college, only 53% completed four or more sessions. Although colleges state that students must maintain a minimum 75% attendance, such was the case in only 2 out of 5 colleges and the low level of attendance in colleges was matched by attendance in CREA-TORS. In addition, some facilitators did not speak the local language, in which some participants preferred to express themselves, for which some facilitators had to be swapped with others. Finally, private colleges permitted only students studying science to participate and all colleges scheduled the program after hours/on weekends, or clashing with classes and examinations, and during severe rain and floods which caused roadblocks in the city.

### 3.1.5 Acceptability

Participants found the intervention highly acceptable in terms of content (97.5%), the format of observing and creating art (97.0%) and the overall experience (98.8%) (see Supplemental Table 2).

Participants found the program’s combination of scientific content about mental health and an arts-based approach as the dominant reasons for high acceptability. They described being able to relate their innermost thoughts and perceptions on mental-health-related stigma through participatory co-creation of art (see Supplemental Table 3 for other examples):

“We don’t (generally) portray issues … Mostly we talk about it or we say this is to be done and this is not to be done, but putting it into art forms, putting it into plays, where you have to show that this is how stereotyped we are and we don’t need to be that (way).” (female, College C)

Another participant shared her thoughts during a session:

“When I saw the posters … I felt astonished and thought - Do these people really have those mental health problems? Whoever we are, we usually feel scared to even sit next to people with mental health issues.” (female, College A)

Participants felt autonomy in decision-making about their art and gained confidence, experiencing a spirit of service to their community, and felt a sense of achievement by putting on an arts show. Such emotions are encapsulated in the following example quotation from a participant:

‘ … that particular time everyone started to talk … People started to put questions, know about it (mental health problems) and started to ask - how can we know … what are the symptoms? … That final workshop somehow made us feel so good, that something that we did reached out to people. So, that was one of the best things.’ (female, College C)

## 4 Discussion

The CREATORS program significantly lowered mental-health-related stigma among participants who created art and had limited non-significant effects on an audience of students observing an arts show, compared to a control group. More than 90% of participants found the program highly acceptable; arts-based activities on the theme of mental-health-related stigma made the subject appealing, accessible, relatable, useful, and interesting to participants. The emphasis on participatory co-creation of art was successful in promoting introspection and sharing ideas on mental health problems by participants and a student audience. Despite the short duration of CREATORS, these elements of the program were able to forge a sense of community among participants.

In our study, we observed positive changes in the intervention groups on knowledge and attitudes but not all items, similar to arts-based interventions having mixed results (Altindag et al., 2006; Gliksman et al., 1983). CREATORS participants’ recognition of symptoms of depression as a mental health problem based on a vignette likely did not improve at post-test because it was at odds with a key message included in their artwork - that only a qualified professional can diagnose depression or other mental disorders (to prevent participants from becoming pseudo psycho-educationists or from labeling one another). Additionally, after researching about mental health problems, it is plausible that some participants believed that mental disorders may be challenging to fully overcome. CREATORS participants’ level of mental-health-related stigma reduced to a greater extent than members of the student audience because the latter received only a brief exposure (arts show). With environmental factors portrayed in the arts show as important determinants of mental health and psychotherapy as treatment, the student audience may not have perceived medication as *the* solution for all mental health problems. These findings are supported by the fact that depression-like symptoms are frequently attributed to socio-economic circumstances in India (Roberts et al., 2020).

### 4.1 Study strengths and implications

#### 4.1.1 A new model of mental-health-related stigma-reduction

The CREATORS program is among the first arts-based interventions to have been developed and piloted, and which reduced mental-health-related stigma among college students in India. We piloted a new model of using youth-focused community-based organization staff, artists, and certified mental health counsellors as facilitators. This model helped to overcome challenges of relying on mental health specialists of whom there is a shortage or involving teachers at understaffed colleges. Mental health specialists often lead mental health promotion in the communities they serve through didactic lectures and may use our arts-based educational methodology and partnership model to conduct activities that may engage youth more effectively.

#### 4.1.2 Age-appropriate engaging content and activities

Given recent efforts to develop and examine the effects of school-based mental health promotion interventions in middle and high schools in India, especially where physical presence of mental health professionals is not feasible (Raman and Thomas, 2023), additional programs to reduce mental-health-related stigma and extend social support during college years may be developed. Similar to students in other arts-based interventions collaborating with clients of mental health services/new people (Twardzicki, 2008) or artists (Salmon et al., 2005), participants in the CREATORS intervention enjoyed and felt excited during rehearsals and live performance.

### 4.1.3 Application in low resource settings

A participating college in our study did not have internet access, a library, any prior externally led program, and students did not have personal mobile phones preventing a mobile-phone/technology-based intervention; CREATORS proved feasible in low-resource settings, and may be potentially applicable in rural areas.

### 4.2 Limitations

This study also has several limitations. Pilot study findings cannot be generalized, predominantly due to the non-randomized convenience sample. Participation was by self-selection; however, participants were not positively predisposed to the theme as fewer workshop participants knew someone with a mental health problem or had past experiences in the arts (included covariates in analysis), and many myths and stereotypes were expressed during sessions. Since no qualitative data were collected among the control group, it remains unclear why there were some changes in knowledge and attitude among this group. In the future, college management’s control over scheduling will be discussed prior to recruitment. Participants at one college may have needed more time to process and discuss distress, fears and suicide prevention strategies since an on-campus suicide (of a non-participant) was reported a day prior to the final arts show. Although a mental health counsellor was available on the day of the final arts show and even discussed participant and audience perceptions about suicide generally, the program may need to include resources and guidance about suicide prevention.

## 5 Conclusions

The CREATORS arts-based program led to a decline in mental-health-related stigma among college youth in India and was an enjoyable and highly acceptable intervention. The program was feasible to implement; participants created an arts show on the theme of mental-health-related stigma to reach other students at their respective colleges as an audience. Application of this program and facilitation model may be tested in other low-and middle-income countries and rural areas with appropriate adaptation, given its successful application in low-resource settings. In the future, a school-based cluster-randomized controlled trial may assess intervention effectiveness.

## Supplementary Material

Supplementary data to this article can be found online at https://doi.org/10.1016/j.ssmmh.2024.100339.

Appendix

## Figures and Tables

**Table 1:** Participant characteristics across study groups, n (%).

	Group 1 Participants creating art (n = 124)	Group 2 Student audience (n = 264)	Group 3 Control Group (n = 86)
**Mean age (S.D.)**	18.79 (1.01)	18.90 (1.86)	18.02 (0.51)
**Age range**	16–21	14–30	17–19
**Gender**			
Male	53 (42.7)	133 (50.4)	27 (31.4)
Female	71 (57.3)	127 (48.1)	59 (68.6)
Missing	0 (0.0)	4 (1.5)	0 (0.0)
**Discipline**			
Science	70 (56.5)	125 (47.4)	0 (0.0)
Social science/Liberal arts	22 (17.7)	131 (49.6)	0 (0.0)
Commerce/Finance	12 (9.7)	0 (0.0)	86 (100.0)
Maths/Computers	18 (14.5)	0 (0.0)	0 (0.0)
Missing	2 (1.6)	8 (3.0)	0 (0.0)
**Year of study**			
1	29 (23.4)	121 (45.8)	86 (100.0)
2	79 (63.7)	69 (26.1)	0 (0.0)
3	8 (6.4)	39 (14.8)	0 (0.0)
4 or more	0 (0.0)	24 (9.1)	0 (0.0)
Missing	8 (6.5)	11 (4.2)	0 (0.0)
**Type of college**			
Government	79 (63.7)	174 (65.9)	0 (0.0)
Private	45 (36.3)	90 (34.1)	86 (100.0)
**Place of origin**			
Hyderabad	73 (58.9)	129 (48.9)	58 (67.4)
Outside Hyderabad	39 (31.4)	99 (37.5)	23 (26.7)
Missing	12 (9.7)	36 (13.6)	5 (5.8)
**Living with parents**			
No (campus/hostel)	34 (27.4)	78 (29.6)	14 (16.3)
Yes	83 (66.9)	163 (61.7)	67 (77.9)
Missing	7 (5.7)	23 (8.7)	5 (5.8)
**Type of previous school**			
Government	53 (42.7)	83 (31.5)	12 (14.0)
Private	67 (54.0)	163 (61.7)	69 (80.2)
Missing	4 (3.3)	18 (6.8)	5 (5.8)
**Cost of transport**			
≤INR 24 per day *(cycle or bus*	60 (48.4)	145 (54.9)	26 (30.2)
≥INR 25 per day *(scooter, car or autorickshaw)*	59 (47.6)	105 (39.8)	53 (61.6)
Missing	5 (4.0)	14 (5.3)	7 (8.2)
**Scholarship eligibility/use**			
Yes	48 (38.7)	124 (46.9)	33 (38.4)
No	64 (51.6)	126 (47.7)	42 (48.8)
Missing	12 (9.7)	14 (5.4)	11 (12.8)
**Participated in extra-curricular (last 3 years)**			
Yes	40 (32.3)	81 (30.7)	46 (53.5)
No	78 (62.9)	170 (64.4)	34 (39.5)
Missing	6 (4.8)	13 (4.9)	6 (7.0)
**Know someone with a mental health problem**			
Yes	43 (34.7)	106 (40.1)	43 (50.0)
No	77 (62.1)	148 (56.1)	39 (45.3)
Missing	4 (3.2)	10 (3.8)	4 (4.7)

**Table 2:** Participants’ knowledge, attitude and behaviors at pre-test and post-test, n (%).

	Group 1 Participants creating art (n = 124)			Group 2 Student audience (n = 264)			Group 3 Control Group (n = 86)		
	Pre-test	Post-test		Pre-test	Post-test		Pre-test	Post-test	
***Knowledge***									
1. Virtually anyone can have poor mental health (Agree)	**101 (81.4)**	**99 (79.8)**		**197 (74.6)**	**198 (75.0)**		**57 (66.3)**	**50 (58.1)**	
2. People with mental health problems are less dangerous than most people think (Agree)	**84 (67.7)**	**88 (70.9)**		**175 (66.2)**	**181 (68.5)**		**57 (66.3)**	**40 (46.5)**	
3. Medication can be an effective treatment for people with mental health problems (Agree)	**84 (67.7)**	**90 (72.6)**		**191 (72.3)**	**185 (70.1)**		**63 (73.2)**	**45 (52.3)**	
4. Identified location of mental health services	**40 (32.3)**	**76 (61.3)**		66 (25.0)	69 (26.1)		**14 (16.3)**	**6 (6.9)**	
5. Recognized symptoms of depression as a mental health problem	**109 (87.9)**	**101 (81.4)**		**184 (69.7)**	**180 (68.2)**		**56 (65.1)**	**40 (46.5)**	
***Attitude***									
6. People with mental health problems are a burden on society (Disagree)	**95 (76.6)**	**97 (78.2)**		**192 (72.7)**	**212 (80.3)**		**59 (68.6)**	**51 (59.3)**	
7. It is best to avoid someone who has a mental health problem (Disagree)	**90 (72.6)**	**81 (65.3)**		**155 (58.7)**	**167 (63.2)**		**55 (63.9)**	**50 (58.1)**	
8. Mental health problems are not real medical illnesses (Disagree)	**38 (30.6)**	**45 (36.3)**		**108 (40.9)**	**66 (25.0)**		**19 (22.1)**	**23 (26.7)**	
9. Mental health problems are a sign of weakness (lack of self-discipline or will power) (Disagree)	**25 (20.2)**	**30 (24.2)**		**93 (35.2)**	**60 (22.7)**		**8 (9.3)**	**10 (11.6)**	
10. People can overcome mental health problems (Agree)	**108 (87.1)**	**107 (86.3)**		**205 (77.6)**	**218 (82.6)**		**71 (82.5)**	**59 (68.6)**	
***Behavior***									
11. Reported and Intended Behavior Scale, mean (S.D.)	**14.79**	**17.15**		**14.59**	**15.50**		12.23	12.40	
	**(3.48)**	**(3.03)**		**(3.59)**	**(3.33)**		(3.56)	(4.03)	
12. Likely to talk about mental health problems of self or others	**96 (77.4)**	**95 (76.6)**		**185 (70.1)**	**194 (73.5)**		**62 (72.1)**	**39 (45.3)**	
13. A friend initiated a conversation/s about mental health	28 (22.6)	55 (44.3)		**67 (25.4)**	**33 (12.5)**		22 (25.6)	15 (17.4)	
14. Initiated a conversation/s about mental health (actual behavior)	**26 (21.0)**	**38 (30.6)**		**36 (13.6)**	**8 (3.0)**		**10 (11.6)**	**12 (13.9)**	

* Likert scale from 1 to 4, where 1 = strongly agree, 2 = slightly agree, 3 = slightly disagree, 4 = strongly disagree. Higher percentages and means at post-test indicate improved knowledge, attitude and intended behavior towards people with mental health problems, i.e., lower mental-health-related stigma. Pre- and post-test values in bold indicate significant differences between pairs using McNemar’s exact test except for Reported and Intended Behavior Scale which was assessed using pairwise comparison of means adjusted using Tukey’s method.

**Table 3:** Multi-level mixed-effects linear regression of Reported and Intended Behavior Scale across three study groups (N = 806).

	Adjusted coefficient [95% Confidence Interval]
*Study group*	
Participants creating art	**2.71 [1.17, 4.24]**
Student audience	**2.61 [1.13, 4.08]**
Control group	Ref
*Time*	
Post-test	0.57 [-0.60, 1.75]
Pre-test	Ref
*Study group x time (between pre-test and post-test)*	
Participants creating art x post-test	**1.55 [0.05, 3.04]**
Student audience x post-test	0.41 [-0.92, 1.75]
Control group x post-test	Ref
*Gender*	
Female	0.23 [-0.24, 0.71]
Male	Ref
*Previous school*	
Private	–0.46 [-1.04, 0.12]
Public	Ref
*Cost of transportation*	
≥INR 25 per day (scooter, car or autorickshaw)	–0.19 [-0.70, 0.30]
≤INR 24 per day (cycle or bus)	Ref
*Previous participation in extra-curricular activities*	
Yes	0.38 [-0.12, 0.88]
No	Ref
*Knowing someone who lives with a mental health problem (at baseline)*	
Yes	**0.85 [0.36, 1.33]**
No	Ref

## Data Availability

De-identified data will be made available upon reasonable request.
