# Supplementary material for: Impact, feasibility, and acceptability of CREATORS: An arts-based pilot intervention to reduce mental-health-related stigma among youth in Hyderabad, India
Source: SSM Ment Health. Author manuscript; Available in PMC 2024 Dec 1. (PMC7616367; doi:10.1016/j.ssmmh.2024.100339)
Supplement: Appendix [file EMS197997-supplement-Appendix.pdf]

Supplemental Table 1. Activity descriptions for six arts-based sessions in the CREATORS program

Program Phase	Session	Activity	Description
Observe	1 Art2Reflect	Visualization	Groups of 6-8 participants use craft materials and paints to illustrate an a person living with a mental health problem.
		Snap	All participants observe posters of real-life people with mental health pr their desire for social distance based on appearances (using post-its ar participants then walk around the room to observe comments.
		TheatreJam	Groups of 6-8 participants develop brief forum theatre scenarios/perforn hypothetical and/or anecdotal distressing situations that could potentiall environmental stressors for mental health problems. All participants car story to change relationships, opinions, communication, body language
	2 Creative Juices	Typecast	All participants play a word association game where they receive a set use to identify/compare/contrast between mental health problems and p problems.
		Inside story	Social contact: All participants interact with a person living with a menta who discusses their life history, experiences of mental-health-related pu present capabilities.
		Ideate	Groups of 6-8 participants brainstorm about how to communicate the ne mental-health-related stigma to their peers. Groups prioritize/select imp and art forms to generate storyboards.
Create	3 Spotlight on Stories	ArtQuiz	A rapid-fire style quiz asking participants (in teams of 6-8 participants) t of artists who created famous paintings and who suffered from mental h well as asking participants to guess different emotions or moods portray selected images. The quiz is designed to recap key messages from ses allow participants to describe art.
		Life-graph	Optional activity: Plot highs and lows in life for the past five years to dis and social support given or received (in pairs)
		Take one	Groups of 6-8 participants working together in Session 2 on Ideate cont concepts, story ideas, and assess resources needed to create art on th
	4 Art4Reach	Amplify	Groups of 6-8 participants create and enact advertisements for less-adv or ideas.
		Kick-start	Groups of 6-8 participants develop advertising plans to invite a student and near-peers to observe an art show.
		Hands-on	Groups of 6-8 participants pitch storyboards/sketches/outlines of their a from peers and facilitators.
Showcase	5 Backstage Bootcamp	Preview	Groups of 6-8 participants rehearse or finalize their artwork. They receiv peers and facilitators to refine messages, artistry and aesthetics, and/or practice.
	6 Arts Show	Showtime	An event to showcase artwork on the theme of mental-health-related pu created by participants with a student audience of peers and near-peers
		Open forum	Participants voluntarily describe their art, share feelings about the arts s discussion, and comments from members of the student audience.

*All activities followed up by discussion on knowledge and stereotypes; optional ice-breaker exercises were used as appropriate

Supplemental Figure 1. Theories and derived program strategies

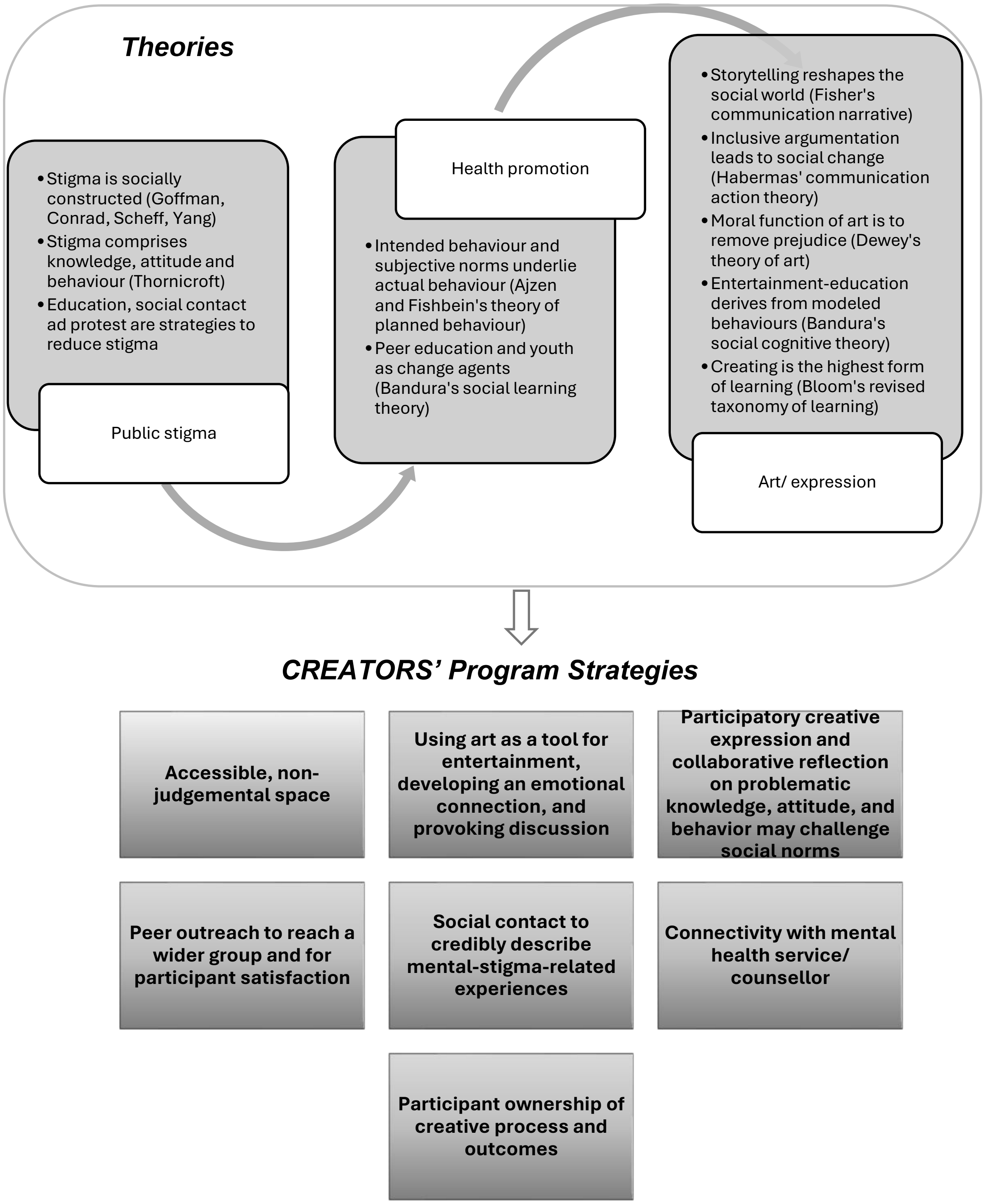

Supplemental Figure 2. Key messages in the CREATORS program

knowledge	1. Physical appearance does not reveal mental health. Signs/ symptoms may not be obvious.
	2. Mental health problems can happen to anyone. Successful and highly productive people also have mental health problems.
	3. Mental health is as important as physical health, and similarly problems should be viewed across a spectrum of severity.
	4.Medication is effective as treatment for mental health problems.
	5. Location of mental health services in the district (hospitals and telephone helpline numbers).
	6. Mental health problems are common.
attitude	7. No one can be blamed for poor mental health.
	8. Mental health does not equal mental illness.
	9. There is no reason to fear a person with a mental health problem, unless their behaviour and thoughts reflect violent sentiments.
	10. Social stigma causes people to hide, delay and even avoid treatment. This damages their life.
intended behaviour	11. Harsh words, insulting and avoiding behaviours can cause distress to anybody, including people with mental health problems. It is hurtful to call someone mental/ mentally unstable or ridicule them.
	12. Talking about mental health problems can make a difference in reducing stigma, and encourage others to talk about problems and seek treatment.
	13. Locking up a person with a mental health problem in a room or any form of isolation, forcibly visiting a faith healer and physical assault is a denial of basic human rights.
	14. Continue social interaction with people facing problems as support and acceptance.

Supplemental Table 2. Participant opinion about CREATORS: an arts-based program aimed at reducing mental-health-related stigma

Acceptability of programme aspects	Agree		Mean (S.D.)*
	%	N^	
Content	**97.52**	**765**	**3.70 (0.47)**
Useful	96.37	771	3.76 (0.56)
Thought-provoking	95.99	773	3.64 (0.59)
Easy to understand	96.62	770	3.71 (0.55)
Format – Art	**97.02**	**773**	**3.58 (0.49)**
Attention and involvement of participants	93.76	768	3.63 (0.65)
Interesting and entertaining	94.06	773	3.65 (0.64)
I was able to share my perception through art	82.60	774	3.25 (0.93)
I felt an emotional connection to the issue	84.63	772	3.22 (0.81)
Experience	**98.84**	**774**	**3.58 (0.45)**
Enjoyable	97.54	772	3.78 (0.51)
Energy/ atmosphere was lively and enthusiastic	95.85	772	3.71 (0.59)
Sufficient opportunity for discussion	92.42	765	3.58 (0.71)
Management of timings and venue	92.77	775	3.59 (0.70)

*Likert scale from 1-4, where 1=strongly disagree, 2= slightly disagree, 3=slightly agree, 4=strongly agree; participant responses as either 3=slightly agree or 4=strongly agree were combined as “Agree.” ^<5% were missing data because participants did not answer the question.

Supplemental Table 3. Illustrative quotations from focus groups demonstrating CREATORS program acceptability

Theme	Illustrative quote
Uniqueness of combining mental-health-related content and arts approach	'I thought it will be (a) common workshop where speeches will be given by the lecturers and professors, which we will have to listen to and then the workshop will get over, but after coming to the class we saw, we experienced and got to learn various art forms and also gained a lot of knowledge from XXX.' (male, College D)
Art as a tool for introspection	'It took me to a different world within a few minutes, which almost made me experience a new me. Because, it's totally different. By this process I learnt something, that's why I like it' (female, College B)
	'You just get stuck thinking about it again and again, and it just gets complicated sometimes if it is a sad thought. So, when we were doing that graph thing I realized how I was thinking at that period of time - I was just stuck with that emotion for a couple of weeks…which was not good.' (female, College C)
Art facilitated reflection on stress and poor mental health	'I imagined the face of my father and then when she (facilitator) said to imagine the same face, but like a person who has a mental health issue - I got all emotional and I got teary-eyed… My father also thinks too much, he has three kids to look after, so thinking about all of us, he also must be having stress inside like what (brother's name) is doing, what (sister's name) is doing and thinking about that, he also might be affected by poor mental health. '(female, College A)
Art challenged perceptions and stereotypes about people living with mental health problems	'When I saw the posters in the workshop, I felt astonished and thought - Do these people really have those mental health problems? Whoever we are, we usually feel scared to even sit next to people with mental health issues.' (female, College A)
Enjoyment from using diverse art forms	'So in XXX they put questions to us and also made us give answers for those, like general questions, then in the between we also had entertainment also and art and in the last week we had skits, songs, dances, acting, so I enjoyed myself a lot. (male, College D)
Autonomy in selecting art forms	'… it was like amalgamation … that's the best form, where you can mix in whatever and you are given the freedom to mix in whatever you want, right? So in the drama itself, we tried to mix in some art along with theatre, so I think that was something that was really enjoyable.' (female, College C)
Art as self-expression	'What I really liked was the fact that we got to express ourselves … so, some of us expressed ourselves through art, some through sculpture, some through words, some through you know painting and drama so I think each person got a chance to do that and that was something very important.' (male, College C)
Motivation to participate – Displaying talent	… in school (like) those particular theater people will be doing it again and again, so here we were not theater people yet we could do it.' (female, College C)
	'before I joined XXX there was a banner in our class and I saw that it had …music, dance, discussion… something that we hadn't done in so long, and we really wanted to you know, get back bits of who we were through that.' (female, College C)

Motivation to participate – Learning about mental health	'I actually joined XXX because I was very interested in doing all arts and stuff… the first time I came was because of this arts and stuff, but then later when I started coming more regularly, because of the mental health issues as we were talking about how every person from any walk of life can become affected with this...' (female, College C)
Participatory co-creation of art through collaboration	I also liked the skit and felt it was useful for me because… when we were told to draw only one person's thoughts are depicted, but in a skit everyone's ideas and actions can be observed, they are incorporated and so I felt that was useful for all.' (male, College B)
Sense of community among participants	'Since this workshop was held once a week we friends shared things, then we also fought over issues, then we had discussions and debates and sometimes also argued as to what is wrong and what is right. So I felt happy being a part of all the workshops...' (female, College A)
	'When we came up in a group, we didn't know any of us. So, a few of our classmates were there, but in a bigger group we were talking about a particular topic, so we were more drawn to that… all of us who were coming for each and every workshop, consequently…we wanted to be in a group, start being in a group and all together work for it so that was one of the things about the workshop.' (female, College C)
Safe space without judgement	'Usually, you won't get to talk about these things without inviting some sort of disdain or some kind of weird looks or saying maybe you should not talk about that, but in this case we were you know, everyone was just so accepting of what we had to say…' (female, College C)
Gained confidence to communicate	'I liked to perform the skit because… due to speaking loudly on (the) stage, my voice has changed and even when I normally speak at home, people will pay attention to what I am saying.' (male, College A)
Social contact	R: On the day when (name of the speaker with a mental health problem) came and told about their life experiences that was interesting… He was an extrovert… He had a mental illness.<br>M: So what struck you about that?<br>R: We thought that the people who are suffering from mental illness they might be different from others, but he seems to be quite normal.' (male, College B)
Sense of pride/achievement based on showcasing art and a positive response from the student audience	'…all that we did there was respected by people and I liked that and felt happy; meaning it was put up in a place that…was clearly visible… felt good that creations that we made, others also could see it.. (female, College A)
	'Doing the play and, by seeing people responding to that - that makes me feel most useful' (male, College B)
	'Everyone was interested in the art work…The audience were asking and we even told the audience how we made it, and what all the artists are saying about it, and they had to see it all.' (R2, male, College B)

Consolidated criteria for reporting qualitative studies (COREQ): 32-item checklist

No. Item	Guide questions/description	Reported on Page # or below
**Domain 1: Research team and reflexivity**		
Personal Characteristics		
1. Interviewer/facilitator	Which author/s conducted the interview or focus group?	9
2. Credentials	What were the researcher's credentials? E.g. PhD, MD	The researcher was a Ph.D. candidate in Public Health at the London School of Hygiene and Tropical Medicine (LSHTM), UK and had completed a Masters in Development Studies (MSc) at the School of Oriental and African Studies, UK.
3. Occupation	What was their occupation at the time of the study?	Ph.D. candidate, LSHTM<br>And<br>Research Associate-Health Communication, Indian Institute of Public Health-Hyderabad, India
4. Gender	Was the researcher male or female?	Female. Pronouns listed on Title Page.
5. Experience and training	What experience or training did the researcher have?	The researcher had spent the previous 6 years as a Research Associate in Health Communication at the Public Health Foundation of India, New Delhi and in Hyderabad, India. The researcher had developed and implemented several pilot community-based and arts-based programs to reduce mental-health-related stigma along with leading the design and implementation of other

		communication campaigns in 7 Indian states. The researcher had previously conducted dozens of focus groups, including among adolescents and related to mental health problems among other sensitive topics. The researcher was trained in Journalism at the undergraduate level (B.A. Honors).
Relationship with participants		
6. Relationship established	Was a relationship established prior to study commencement?	No
7. Participant knowledge of the interviewer	What did the participants know about the researcher? e.g. personal goals, reasons for doing the research	Participants knew that the researcher was interested in understanding their experience and engagement with the program and their opinion about different program elements.
8. Interviewer characteristics	What characteristics were reported about the interviewer/facilitator? e.g. Bias, assumptions, reasons and interests in the research topic	The facilitator also co-developed the program and was interested to examine its effects (program benefits, harms, strengths, and weaknesses) among young people. Previous involvement in piloting arts-based educational interventions did not include any quantitative assessment.

**Domain 2: study design**		
Theoretical framework		
9. Methodological orientation and Theory	What methodological orientation was stated to underpin the study? e.g. grounded theory, discourse analysis, ethnography,	9

	phenomenology, content analysis	
Participant selection		
10. Sampling	How were participants selected? e.g. purposive, convenience, consecutive, snowball	5
11. Method of approach	How were participants approached? e.g. face-to-face, telephone, mail, email	5; Face-to-face
12. Sample size	How many participants were in the study?	<table><tr><td>College</td><td>Number of participants</td></tr><tr><td>A</td><td>12</td></tr><tr><td>B</td><td>11</td></tr><tr><td>C</td><td>9</td></tr><tr><td>D</td><td>10</td></tr><tr><td>Total</td><td>42</td></tr></table>
13. Non-participation	How many people refused to participate or dropped out? Reasons?	None dropped out of the focus groups
Setting		
14. Setting of data collection	Where was the data collected? e.g. home, clinic, workplace	On college campuses (outdoor space, classroom, and canteen during off peak hours)
15. Presence of non-participants	Was anyone else present besides the participants and researchers?	No
16. Description of sample	What are the important characteristics of the sample? e.g. demographic data, date	59.5% - Female<br>Participants reported engagement in multiple aspects of the CREATORS program:<br>40.4% - Visual art (17)<br>35.7% - Theater (16)<br>26.1% - Dance (11)<br>10.0% - Setup (8)<br>4.7% - Those who dropped out mid-program and joined focus groups (2)
Data collection		
17. Interview guide	Were questions, prompts, guides provided by the authors? Was it pilot tested?	A focus group discussion guide is attached below. These questions were pilot

		tested for the first time during this study.
18. Repeat interviews	Were repeat interviews carried out? If yes, how many?	No repeat focus groups were conducted.
19. Audio/visual recording	Did the research use audio or visual recording to collect the data?	Only audio recording.
20. Field notes	Were field notes made during and/or after the interview or focus group?	Yes
21. Duration	What was the duration of the interviews or focus group?	One hour
22. Data saturation	Was data saturation discussed?	9
23. Transcripts returned	Were transcripts returned to participants for comment and/or correction?	No. During focus groups clarifications and comments were invited.
**Domain 3: analysis and findings**		
Data analysis		
24. Number of data coders	How many data coders coded the data?	Two; First author and field research assistant
25. Description of the coding tree	Did authors provide a description of the coding tree?	No
26. Derivation of themes	Were themes identified in advance or derived from the data?	9
27. Software	What software, if applicable, was used to manage the data?	9
28. Participant checking	Did participants provide feedback on the findings?	No. During focus groups clarifications and comments were invited.
Reporting		
29. Quotations presented	Were participant quotations presented to illustrate the themes/findings?<br>Was each quotation identified? e.g. participant number	Page 13-14 and Supplemental Table 3.<br>Yes, by gender and college.
30. Data and findings consistent	Was there consistency between the data presented and the findings?	Pages 12, 13, and 14 and in Supplemental Table 2 and 3.
31. Clarity of major themes	Were major themes clearly presented in the findings?	Higher-level theme on acceptability on Pages 12-14. Major and minor themes in Supplemental Table 3.
32. Clarity of minor themes	Is there a description of diverse cases or discussion of minor themes?	Supplemental Table 3

FOCUS GROUP TOPIC GUIDE AND PROBES

MODERATED BY RESEARCH COORDINATOR (PhD student) and Field Research Assistant (where participants preferred to use the local language)

INTRODUCTION

Welcome to our discussion group and thank you for joining us today. My name is Shivani Mathur Gaiha and I am a PhD student at the London School of Hygiene and Tropical Medicine, UK. I am also serving as a Research Associate at the Indian Institute of Public Health-Hyderabad, a regional branch of the Public Health Foundation of India.

PURPOSE

I am conducting these discussions to understand your thoughts and opinions about the arts-based programme that you participated in for the last six weeks. Your views are very important, even if you feel that you are unsure about what to say, so please feel free to share what comes to mind during the discussion. The results of this study will provide data that may guide the development of workshops in the future.

CONFIDENTIALITY

During the discussion, … will be writing down notes, and reminding me if I forget to ask something. I will be recording our conversation using a digital audio-recorder. Please do not be concerned about this, as all notes, audio files and transcripts of the same will be will not be shared with individuals outside of the research team and any quotations used in any publication will be anonymized. It is only for my reference as I am likely to be unable to note all your comments down.

INFORMED CONSENT

If you agree to participate, please read and sign the consent form (pass out consent forms). Your decision to participate is completely voluntary – you can withdraw from the study at any time without explanation and without consequence. You will also have the right to refuse to answer any question that is asked of you, and ask that any data you have supplied be withdrawn/ destroyed. (Ensure that everyone fills and signs consent form – details collected include names, gender, college, district, date, date of birth and signature).

GROUP RULES
Now I will go over some rules for our discussion:
1. This is not a test, so there are no right or wrong answers. Your participation here will not be graded or scored or count towards attendance at college.
2. It is important that only one person speaks at a time. We will not go around the group, so just join in whenever you want to say something, after the previous speaker has finished their point. Whoever is speaking will be given full attention and respect, so we request you to have no side conversations.
3. Everything said here is confidential and I would urge you not to share it beyond this discussion.
4. Every opinion counts, so it is alright for you to disagree with everyone else. Please come forward as we are interested in everyone's ideas and hope that you will give each other the

opportunity to fully participate. The discussion will take approximately 1 hour. Are there any questions before we start? Let's begin!

OPENING QUESTION As an introduction, let's go around the group so each of you can introduce yourselves. Perhaps you could tell us your name and in one word describe the last six weeks.

DISCUSSION QUESTIONS & PROBES
Now I would like to ask the group some questions.
1. What made you join this program? Probe: • What appealed to you? What motivated you? • What was your expectation?

2. How was mental health viewed amongst your friends at the start of the program? Probes: • What shapes/ contributed to this perception? In real life/ media?

3. How did you find the experience of taking part? Probes: • Can you describe how you felt as part of the group? • What did you like or dislike? • How did you see your role in the process? How involved did you feel? • Did the program meet your expectation?

4. What did you find useful about the program? Probes: • Are there any particular sessions/ exercises that you feel were interesting? Why? • What did you think of the topic? Is it relevant to your life?

5. Has the program made any difference to your knowledge of mental health? Probes: • Which session made the maximum difference?

6. Has the program made any difference to your attitude towards mental health? Probes: • Can you describe your emotions during the program? • Which session made the maximum difference? How?

7. What was the response of the audience/ peer observers' to the Creative Arts Showcase? Probes: • Challenges in inviting peer observers/ audience for the Creative Arts Showcase? Did their attitude have some effect on their interest? • Has the program made a difference to talking about mental health with your friends? • Perceived opinion on content, art work and discussion • Did anyone come up to you after the show? What did they say?

8. Overall, what is your opinion of the arts-based mental health promotion program? Probes: • What do you feel it achieved? • What are its strengths and weaknesses? • What would you suggest to improve the program?

CONCLUSION
Summarize main points from discussion. We are now reaching the end of our discussion. Does anyone have any further comments to add before we conclude the session? Does anyone have any questions? If anyone wants to change or remove any comment made by them during the discussion, you can ask to do it now or contact us at the number provided in the participant information sheet at a later time. We thank you very much for your time and opinions!

STROBE Statement—Checklist of items that should be included in reports of ***cohort studies***

	Item No	Recommendation	Page No
**Title and abstract**	1	(*a*) Indicate the study's design with a commonly used term in the title or the abstract<br>(*b*) Provide in the abstract an informative and balanced summary of what was done and what was found	1-2
**Introduction**			
Background/rationale	2	Explain the scientific background and rationale for the investigation being reported	3-5
Objectives	3	State specific objectives, including any prespecified hypotheses	5
**Methods**			
Study design	4	Present key elements of study design early in the paper	5
Setting	5	Describe the setting, locations, and relevant dates, including periods of recruitment, exposure, follow-up, and data collection	5-9
Participants	6	(*a*) Give the eligibility criteria, and the sources and methods of selection of participants. Describe methods of follow-up<br>(*b*) For matched studies, give matching criteria and number of exposed and unexposed	5<br>n/a
Variables	7	Clearly define all outcomes, exposures, predictors, potential confounders, and effect modifiers. Give diagnostic criteria, if applicable	7-9
Data sources/ measurement	8*	For each variable of interest, give sources of data and details of methods of assessment (measurement). Describe comparability of assessment methods if there is more than one group	7-9
Bias	9	Describe any efforts to address potential sources of bias	9, 16
Study size	10	Explain how the study size was arrived at	16
Quantitative variables	11	Explain how quantitative variables were handled in the analyses. If applicable, describe which groupings were chosen and why	7-8
Statistical methods	12	(*a*) Describe all statistical methods, including those used to control for confounding<br>(*b*) Describe any methods used to examine subgroups and interactions<br>(*c*) Explain how missing data were addressed<br>(*d*) If applicable, explain how loss to follow-up was addressed<br>(*e*) Describe any sensitivity analyses	9<br>7,9<br>12<br>n/a<br>n/a
**Results**			
Participants	13*	(a) Report numbers of individuals at each stage of study—eg numbers potentially eligible, examined for eligibility, confirmed eligible, included in the study, completing follow-up, and analysed<br>(b) Give reasons for non-participation at each stage<br>(c) Consider use of a flow diagram	10 (numbers included, completing follow-up, and analyzed)<br>12<br>N/a in this convenience sample

Descriptive data	14*	(a) Give characteristics of study participants (eg demographic, clinical, social) and information on exposures and potential confounders	10; Table 1
		(b) Indicate number of participants with missing data for each variable of interest	Table 1
		(c) Summarise follow-up time (eg, average and total amount)	7
Outcome data	15*	Report numbers of outcome events or summary measures over time	10-11

Main results	16	(*a*) Give unadjusted estimates and, if applicable, confounder-adjusted estimates and their precision (eg, 95% confidence interval). Make clear which confounders were adjusted for and why they were included<br>(*b*) Report category boundaries when continuous variables were categorized<br>(*c*) If relevant, consider translating estimates of relative risk into absolute risk for a meaningful time period	8,10<br>Table 3<br>n/a
Other analyses	17	Report other analyses done—eg analyses of subgroups and interactions, and sensitivity analyses	10-12; Table 3
**Discussion**			
Key results	18	Summarise key results with reference to study objectives	14
Limitations	19	Discuss limitations of the study, taking into account sources of potential bias or imprecision. Discuss both direction and magnitude of any potential bias	16
Interpretation	20	Give a cautious overall interpretation of results considering objectives, limitations, multiplicity of analyses, results from similar studies, and other relevant evidence	14-16
Generalisability	21	Discuss the generalisability (external validity) of the study results	16
**Other information**			
Funding	22	Give the source of funding and the role of the funders for the present study and, if applicable, for the original study on which the present article is based	Title Page
